# Molecular Subtypes of Glioblastoma Are Relevant to Lower Grade Glioma

**DOI:** 10.1371/journal.pone.0091216

**Published:** 2014-03-10

**Authors:** Xiaowei Guan, Jaime Vengoechea, Siyuan Zheng, Andrew E. Sloan, Yanwen Chen, Daniel J. Brat, Brian Patrick O’Neill, John de Groot, Shlomit Yust-Katz, Wai-Kwan Alfred Yung, Mark L. Cohen, Kenneth D. Aldape, Steven Rosenfeld, Roeland G. W. Verhaak, Jill S. Barnholtz-Sloan

**Affiliations:** 1 Case Comprehensive Cancer Center, Case Western Reserve University School of Medicine, Cleveland, Ohio, United States of America; 2 Department of Internal Medicine, University Hospitals Case Medical Center, Cleveland, Ohio, United States of America; 3 The University of Texas MD Anderson Cancer Center, Houston, Texas, United States of America; 4 Department of Neurological Surgery, University Hospitals Case Medical Center, Cleveland, Ohio, United States of America; 5 Department of Pathology and Laboratory Medicine, Emory University School of Medicine, Atlanta, Georgia, United States of America; 6 Department of Neurology, Mayo Clinic, Rochester, Minnesota, United States of America; 7 Department of Pathology, University Hospitals Case Medical Center, Cleveland, Ohio, United States of America; 8 Cleveland Clinic, Cleveland, Ohio, United States of America; Beijing Tiantan Hospital, Capital Medical University, China

## Abstract

**Background:**

Gliomas are the most common primary malignant brain tumors in adults with great heterogeneity in histopathology and clinical course. The intent was to evaluate the relevance of known glioblastoma (GBM) expression and methylation based subtypes to grade II and III gliomas (ie. lower grade gliomas).

**Methods:**

Gene expression array, single nucleotide polymorphism (SNP) array and clinical data were obtained for 228 GBMs and 176 grade II/II gliomas (GII/III) from the publically available Rembrandt dataset. Two additional datasets with *IDH1* mutation status were utilized as validation datasets (one publicly available dataset and one newly generated dataset from MD Anderson). Unsupervised clustering was performed and compared to gene expression subtypes assigned using the Verhaak et al 840-gene classifier. The glioma-CpG Island Methylator Phenotype (G-CIMP) was assigned using prediction models by Fine et al.

**Results:**

Unsupervised clustering by gene expression aligned with the Verhaak 840-gene subtype group assignments. GII/IIIs were preferentially assigned to the proneural subtype with *IDH1* mutation and G-CIMP. GBMs were evenly distributed among the four subtypes. Proneural, *IDH1* mutant, G-CIMP GII/III s had significantly better survival than other molecular subtypes. Only 6% of GBMs were proneural and had either IDH1 mutation or G-CIMP but these tumors had significantly better survival than other GBMs. Copy number changes in chromosomes 1p and 19q were associated with GII/IIIs, while these changes in *CDKN2A*, *PTEN* and *EGFR* were more commonly associated with GBMs.

**Conclusions:**

GBM gene-expression and methylation based subtypes are relevant for GII/III s and associate with overall survival differences. A better understanding of the association between these subtypes and GII/IIIs could further knowledge regarding prognosis and mechanisms of glioma progression.

## Introduction

Brain tumors contribute to a disproportionate share of cancer-associated morbidity and mortality. Gliomas, graded from II to IV according to The World Health Organization (WHO), [1] are the most common types of primary malignant brain tumors in adults. The grade IV glioblastoma (GBM) is one of the most lethal cancers, with a two-year survival rate around 25%. [2], [3] Grade II/III gliomas (GII/III) overall have longer survival but ultimately transform to a higher grade tumor, with greater mortality. [4]–[6] In clinical practice gliomas are assessed and graded by pathologists based on histological features that are subject to inter-observer variability which could lead to ambiguous diagnosis for some patients. [7]–[9].

Genomic profiling can help to circumvent histopathological diagnosis limitations by using genetic, epigenetic and transcriptomic data as aids to more objectively stratify brain tumors. [10]–[13] Multiple studies have utilized these types of genomic data for brain tumor stratification, for example, higher grade gliomas (grades III and IV) were divided into three groups based on their association with clinical outcome. [12] Using a larger cohort of GBMs, The Cancer Genome Atlas (TCGA) project used an unsupervised approach that led to the classification of the GBMs into proneural, neural, classical and mesenchymal gene expression based subtypes. [14] Importantly, a subset of the proneural GBMs was later found to present a glioma associated CpG Island Methylator Phenotype (G-CIMP) and was tightly tied to the R132 mutation in *IDH1*. [13] Mechanistic studies found that this mutation produced 2-hydroxyglutarate and remodels the methylome. [15]–[17] The R132 *IDH1* mutation, which was first reported in GBM, [18] is a prevalent event in lower grade gliomas and is a prognostic marker for better prognosis in both GII/IIIs and GBMs. [18]–[21] The better survival of GII/IIIs, especially those with the proneural subtype, has been attributed in large part to the distinctive genetic and clinical characteristics of *IDH1* mutant tumors. [19] These studies collectively advanced our understanding of molecular stratification of gliomas.

Similar subgrouping efforts based on genomic data for GII/IIIs have lagged behind, possibly due to the lower population incidence of these tumors as compared to GBMs. GII/IIIs represent an ensemble of diseases, including oliodendroglioma, astrocytoma and olioastrocytoma (also called mixed gliomas). This classification paradigm is mainly owing to the morphological resemblance of tumor cells to glial cells. Besides the recent finding of the R132 *IDH1* mutation, 1p/19q co-deletion is considered a favorable prognostic factor for GII/IIIs. [22] However, the association between genome-wide classifiers and clinical features of these gliomas is still unclear.

To address this problem, we collected more than 700 glioma gene expression profiles from datasets in the public domain and newly generated datasets from our own efforts to study the association between known molecular subtypes of GBM with GII/IIIs, including gene expression subtypes and *IDH1/*G-CIMP statuses. Our results unveiled shared patterns between GII/IIIs and GBMs, suggesting common molecular features between grades of gliomas.

## Materials and Methods

### Datasets and Normalization

#### DASL Dataset (newly generated dataset)

Tumor samples from 144 glioma patients were prospectively collected and processed (formalin fixed and paraffin embedded [FFPE]) at MD Anderson Cancer Center, with prior approval from the MD Anderson Cancer Center Institutional Review Board. Expression profiles were generated using the Illumina cDNA-mediated Annealing, Selection, extension, and Ligation (DASL) Assay protocol. Low level summary signals were extracted from the arrays using the *beadarray* R package. [23] Quantile normalization was applied and probe signals were collapsed to gene levels using the maximal values. Mutational status was assessed on 184 pre-selected mutations, including *IDH* R132, using the Sequenom platform. The final DASL dataset consisted of 141 patients with annotated clinical information, of which 115 have a known *IDH1* mutation status. These data have been deposited into the Gene Expression Omnibus (GEO) under *GSE54004.*

#### Rembrandt Dataset (publicly available dataset)

Raw gene expression (Affymetrix U133 Plus 2.0), SNP array (Affymetrix 100K) and clinical data were acquired from the publically available Repository for Molecular Brain Neoplasia Data (Rembrandt) (https://caintegrator.nci.nih.gov/rembrandt/), which included data on 228 GBMs and 215 GII/IIIs. The histological grade (II vs. III) was available for 176 GII/IIIs, as derived from caArray (http://array.nci.nih.gov). Hence, the overall Rembrandt gene expression dataset consisted of 404 Grade II-IV glioma patients. A set of 334 Rembrandt Affymetrix 100K SNP array samples from 23 oligo II tumors, 21 oligo III tumors, 57 astro II tumors, 45 astro III tumors and 188 GBM tumors and paired normal samples was obtained. The raw gene expression files and SNP array files were processed using the same procedures as described in Verhaak et al. [11] Samples with mixed subtypes were removed due to a very small sample size.

#### JCO Dataset (publically available dataset)

A pool of 853 samples was sequenced for *IDH1* status (cohorts A-H, excluding cohort I: 150, referred to as the JCO data set). [24] Among them, 171 samples were successfully classified into gene expression based subtypes established by Verhaak et al [11], by pooling data from raw gene expression obtained from the authors, and gene expression data obtained from the GEO database (GSE4271). Pathology examination categorized these samples into 150 GBMs and 21 grade III astrocytomas. R132 *IDH1* mutation information and annotated clinical information was gathered from corresponding supplementary files. 171 samples with matching gene expression data and *IDH1* status were used for analysis. [24].

In total, a pool of 716 glioma samples was used for overall analysis: 71 astrocytoma grade II (Astro II), 105 astrocytoma grade III (Astro III), 35 oligodendroglima II (Oligo II), 29 oligodendroglioma III (Oligo III) and 476 GBM (Table 1). R132 *IDH1* mutation status was available for 286 gliomas (from DASL and JCO) and was used as a proxy for G-CIMP status. Where *IDH1* or G-CIMP status was not available, the status was predicted using gene expression data (see section below for further details). Survival information was available for 617 patients (from Rembrandt, DASL and JCO) including 55 Astro II, 89 Astro III, 24 Oligo II, 27 Oligo III and 422 GBM. The University hospitals of Cleveland IRB approved this research as exempt, and MD Anderson Cancer Center IRB approved the DASL samples mentioned previously.

**Table 1:** Clinical information and median survival by gene expression subtype and histological group for overall study combined dataset (n = 404 Rembrandt+171 JCO+141 DASL = 716 TOTAL).

		Classical (N = 135)	Mesenchymal (N = 149)	Neural (N = 89)	Proneural (IDH1-/NON G-CIMP) (N = 74)	Proneural (IDH1+/G-CIMP) (N = 28)
**GBM (N = 476)**	**Average Age** *	57.66	55.36	54.36	55.49	43.68
	**Survival (p = 2.05e-09)** *	19 (15.8, 27.5)	22.8 (19.6, 29.5)	26.2 (20.8, 32.4)	23.9 (19.3, 32.2)	48.3 (28.5, NA)
	**Race (White%)&**	41(95.3%)	44(95.7%)	39(97.5%)	24(100%)	10(90.9%)
	**Gender (Male%)** *	64 ( 56.1 %)	95 ( 71.4 %)	50 ( 62.5 %)	43(68.25%)	14(50%)
		**Classical (N = 3)**	**Mesenchymal (N = 17)**	**Neural (N = 12)**	**Proneural (IDH1-/NON G-CIMP) (N = 1)**	**Proneural (IDH1+/G-CIMP) (N = 37)**
**Astro II (N = 71)**	**Average Age** *	38.67	46.12	41.8	57	38.14
	**Survival (p = 7.17e-04)** *	NA (9, NA)	44.6 (15.2, NA)	56.6 (43.1, NA)	NA (13.7, NA)	115.7 (76.8, NA)
	**Race (White%)^&^**	1(50%)	11(91.67%)	3(60%)	1(100%)	16(100%)
	**Gender (Male%)** *	1(50%)	10(76.92%)	7(100%)	1(100%)	17(58.62%)
		**Classical (N = 18)**	**Mesenchymal (N = 17)**	**Neural (N = 17)**	**Proneural (IDH1-/NON G-CIMP) (N = 6)**	**Proneural (IDH1+/G-CIMP) (N = 45)**
**Astro III (N = 105)**	**Average Age** *	54.67	43.27	43.59	36.5	36.98
	**Survival (p = 2.35e-04)** *	27.6 (21.2, NA)	42.5 (15.8, NA)	40.8 (28.1, NA)	109 (59.4, NA)	148.9 (78.8, NA)
	**Race (White%)&**	10(100%)	8(72.73%)	4(44.44%)	2(40%)	15(60%)
	**Gender (Male%)** *	4 ( 33.3 %)	8 ( 57.1 %)	9 ( 60 %)	5(100%)	26(60.47%)
		**Classical (N = 1)**	**Mesenchymal (N = 5)**	**Neural (N = 10)**	**Proneural (IDH1-/NON G-CIMP) (N = 1)**	**Proneural (IDH1+/G-CIMP) (N = 17)**
**Oligo II (N = 35)**	**Average Age** *	57	52	46.5	62	44.57
	**Survival (p = 1.0e-02)** *	NA (NA, NA)	NA (19.6, NA)	45.3 (14.4, NA)	NA (NA, NA)	NA (NA, NA)
	**Race (White%)&**	1(100%)	1(50%)	6(100%)	0	9(90%)
	**Gender (Male%)** *	1 ( 100 %)	3 ( 100 %)	2 ( 28.6 %)	1(100%)	4 ( 30.8 %)
		**Classical (N = 4)**	**Mesenchymal (N = 5)**	**Neural (N = 2)**	**Proneural (IDH1-/NON G-CIMP) (N = 4)**	**Proneural (IDH1+/G-CIMP) (N = 14)**
**Oligo III (N = 29)**	**Average Age** *	55.75	48	37	42	42.14
	**Survival (p = 4.6e-01)** *	12 (8.8, NA)	30.8 (10.7, NA)	6.4 (6.4, NA)	34.1 (22.3, NA)	81.5 (41.9, NA)
	**Race (White%)&**	2(100%)	3(100%)	1(100%)	3(100%)	7(100%)
	**Gender (Male%)** *	1 ( 50 %)	1 ( 33.3 %)	0 ( 0 %)	2(66.7%)	9 ( 64.3 %)

* Included in all three dataset.

Median survival in months (95% CI) was adjusted for age. P values were reported using Log-rank test.

&Included in Rembrandt only.

**Figure 1:**
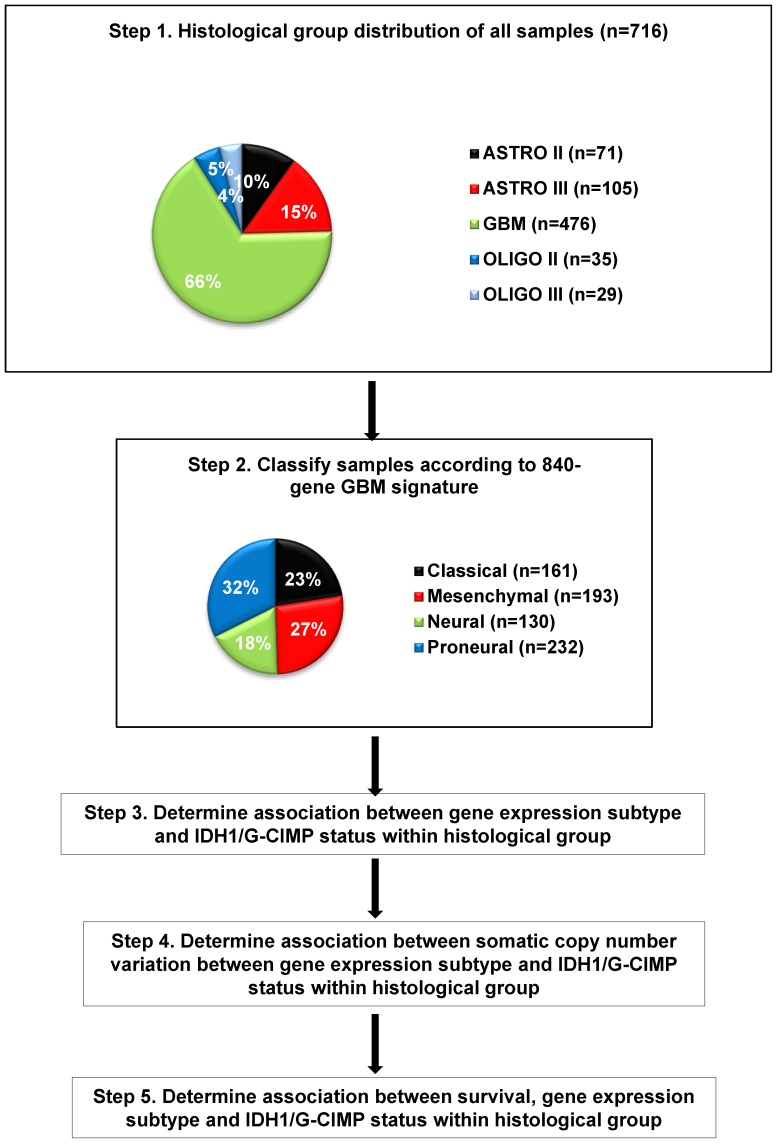
Overall analysis approach.

The general overall analysis approach is outlined in Figure 1.

### Gene expression analysis

Unsupervised clustering of the Rembrandt data was performed by filtering expression profiles to select the top 1,500 variable genes, and the resulting data set was subjected to non-negative matrix factorization clustering. In this process, random subsets of the data are clustered many times to identify the most robust clusters.

The four gene signatures as established by Verhaak et al were projected onto the Rembrandt data using the single sample gene set enrichment analysis (ssGSEA) [25], [26] method. ssGSEA obtains an enrichment score by an integration of the difference between the empirical cumulative distribution functions (ECDFs) of genes in the gene set and the remainder of the genes in one expression profile, after ranking genes by absolute expression level. The ssGSEA method was then used to assign a “gene expression subtype” to the Rembrandt, JCO and DASL datasets. Then, Fisher’s exact test was used to determine potential overlap between the results of unsupervised clustering and ssGSEA.

### Identification of G-CIMP positive cases for the Rembrandt dataset and generation of *IDH1*/G-CIMP status

The Noushmehr et al [13] glioma-CpG Island Methylator Phenotype (G-CIMP) predictions were generated as a surrogate of R132 *IDH1* mutation status for the Rembrandt data set using the available gene expression array data. Five sets of probes predictive of G-CIMP status (10-probe, 25-probe, 50-probe, 100-probe, and 200-probe) [27] were used to compute G-CIMP status, and a consensus of five sets of G-CIMP predictions was extracted via nearest neighbors algorithm according to the method by Fine et al. [27] K-means consensus clustering with 1000 iterations, random start and Euclidean distance metric for sample ordering was applied to the rest of Rembrandt data using the same five sets of predictors, and consensus G-CIMP predictions of those sets were determined. A new variable that unified G-CIMP predictions and R132 *IDH1* mutation status was generated (*IDH1*/G-CIMP).

### DNA copy number analysis

To identify somatic copy number alterations (SCNAs), Genomic Identification of Significant Targets in Cancer (GISTIC) [28] with default parameters via GenePattern platform [29] was used. GISTIC identifies both focal and broad SCNA events that were used to investigate associations via SCNAs and histological groups, *IDH1*/G-CIMP status and gene expression subtypes using a two sided Fisher’s exact test.

### Survival analysis

Kaplan-Meier (KM) survival analysis was performed to compare the survival of patients among five molecular subtype groups by histological group and by grade; the five molecular subtype groups were Classical, Mesenchymal, Neural, *IDH1+/*G-CIMP (defined as proneural and *IDH1* mutant) and *IDH1-*/non G-CIMP tumors (defined as proneural and *IDH1* wildtype). The log rank test was used to test for survival differences amongst the molecular subtype groups. Cox regression survival analysis was used in order to adjust for age at diagnosis (generating age adjusted median survival estimates with 95% confidence intervals) and/or gene expression based subtype group and differences in survival amongst the molecular subtype groups were visualized using Kaplan-Meier survival analysis. Survival time was censored for those living greater than 60 months in order to be comparable to other studies. [11] The average follow-up in Rembrandt was 4 years, with a range from 0 to 20 years.

## Results

To validate the presence of the proneural/neural/classical/mesenchymal expression subtypes in lower grade gliomas we used data from two publically available datasets that we refer to as Rembrandt and JCO, respectively; [24] and generated gene expression data from 141 new formalin fixed, paraffin embedded (FFPE) gliomas using the DASL platform. This data set represented all the common grades and histologies of glioma, including 64 oligodendrogliomas (grade II: 35, grade III: 29), 176 astrocytomas (grade II: 71, grade III: 105) and 476 glioblastoma for a combined total of 716 samples. Figure 1 outlines the overall analysis approach and results from each step are outlined in the following sections.

### Rembrandt derived expression subtypes resemble Verhaak GBM expression classes

To identify the factors that drive the gene expression based clustering of low and high grade glioma across histologies, we began by using the Rembrandt dataset and performed unsupervised non-negative matrix factorization clustering using the 1,500 most variable genes, and divided the 404 samples into four clusters. Although some preference of histology for a certain cluster was observed (i.e. 46% of GBM were found in cluster 1), each cluster was heterogeneous and included all histology types. When comparing the results from unsupervised clustering to the four subtype classification suggested by Verhaak et al using the 840-gene expression based signature, we found a highly significant overlap (*P* = 9.88×10^−1^, Fisher’s Exact Test for lack of overlap) (Table S1 and Figure S1), although this result could have been influenced by the inclusion of GBMs from Rembrandt. Of the 1500 genes we used in the unsupervised analysis, only 239 (15.9%) were present on the 840 gene list, suggesting that the clustering overlap was robust and not caused by the gene overlap. As the unsupervised clustering largely confirmed the applicability of the GBM subtypes in GII/IIIs, we further focused on the subtypes as predicted by the 840-gene expression based signature.

The percentages per subtype amongst the GBM in our combined data set (N = 457) mirrored the distribution from the original TCGA GBM report [11] (Fisher’s exact test *P* =  2.13×10^−1^ for distributional differences between estimation techniques). However, the proneural subtype with *IDH1* mutation was more prevalent among GII/IIIs compared with GBMs, seen in 53% of tumors across histological subtypes, as contrasted with only 24% of GBMs. The classical signature was almost absent among Astro II tumors (4%) and more frequent in Oligo III (14%) (Table S2).

### G-CIMP predictions or IDH1 mutation status

The distribution of proneural in GBMs with respect to *IDH1+*/G-CIMP status was opposite to that observed in GII/IIIs. In the Rembrandt dataset, 64% of tumors were predicted to be G-CIMP negative. The majority of these were GBMs. For JCO and DASL, R132 *IDH1* mutation was negative in 82% and 78% of the total patients in each dataset, respectively. Comparing percentage distributions of G-CIMP negative in Rembrandt with *IDH1* mutation negative in JCO and DASL, there were no significant differences between DASL and Rembrandt or between DASL and JCO, with Fisher’s exact test *P* values of 4.28×10^−1^ and 7.75×10^−2^ respectively. However, there was a significant difference in percentage distributions between Rembrandt and JCO, with a Fisher’s exact test p value of 6.50×10^−3^ (Table S3), most likely due to the proportional differences between these datasets in terms of histological groups represented. In the combined dataset few GBMs were *IDH1*+/G-CIMP, compared to approximately 50% of the GII/IIIs.

**Figure 2:**
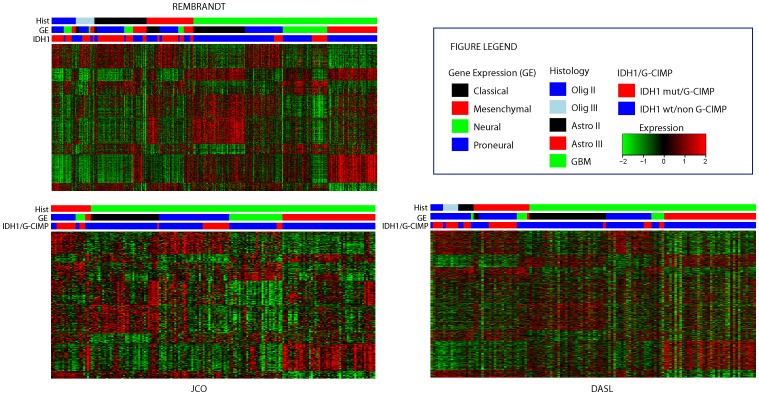
Heatmaps for the Verhaak 840-gene expression based subtype by histological group and IDH1/G-CIMP status in the Rembrandt, JCO and DASL datasets.

Figure 2 shows gene expression heatmaps for the 840-gene expression based signature in each of the three datasets, according to histological subtype and presence or absence of an *IDH1* mutation or G-CIMP. The gene expression subtypes can be distinguished within each histological subtype, and tumors within the same gene expression subtype have similar expression patterns irrespective of their histological subtype or dataset. However, the presence of an *IDH1* mutation is associated with a different expression pattern, as most of proneural tumors had an *IDH1* mutation and vice versa, compared to that of classical tumors (Table S4). For instance, in the DASL data set, 32 of 35 cases with *IDH1* R132 mutation were classified as Peroneural. The high correlation of *IDH1* mutation and the Proneural subtype not only confirmed our previous report in GBM [11], but also illustrated the quality of our data set. This conclusion is best appreciated in the proneural GBMs in any of the datasets where a much lower proportion of GBMs have *IDH1*+/G-CIMP as compared to the GII/IIIs.

### Survival distribution

**Figure 3:**
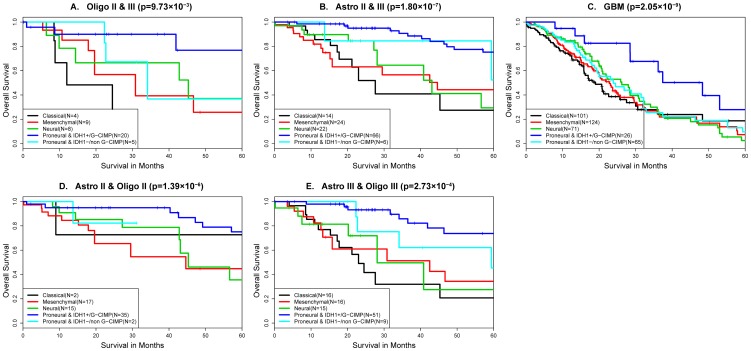
Survival analysis of gene expression subtype and *IDH1*/G-CIMP status by histological group and by grade of tumor adjusted for age at diagnosis. Merged dataset of JCO, Rembrandt and DASL (A: Oligo II& III (N = 46); B: Astro II & III (N = 132); C: GBM (N = 387); D: Grade II(N = 71); E: Grade III (N = 107)).

Associations between gene expression subtype and patient outcome have been previously described. [12] We aimed to evaluate the association between the five molecular subtypes (proneural and *IDH1* mutant, proneural and *IDH1* wildtype, neural, classical, mesenchymal) and overall survival, using samples for which survival annotation was available from the JCO, Rembrandt and DASL datasets (Figure 3A, B, C, oligodendrogliomas: N = 46, astrocytomas: N = 132 and GBM: N = 387)). Survival analysis was performed by grouping all tumors according to histological type and separately by grouping tumors according to grade (Figure 3D, E, C, Grade II: N = 71, Grade III: N = 107 and GBM: N = 387)). Across all histological types, proneural *IDH1*+/G-CIMP tumors had the better survival. (Figure 3A: *P* = 9.73×10^−3^, 3B: *P* = 1.80×10^−7^, 3C: *P* = 2.05×10^−9^). Proneural *IDH1*-/non G-CIMP tumors had a survival comparable to that of other expression subtypes. Similar observations were found when analyzing according to grade of tumor (Figure 3D: *P* = 1.39×10^−6^, 3E: *P* = 2.73×10^−4^, 3C: *P* = 2.05×10^−9^).

### Somatic copy number alteration analysis

**Figure 4:**
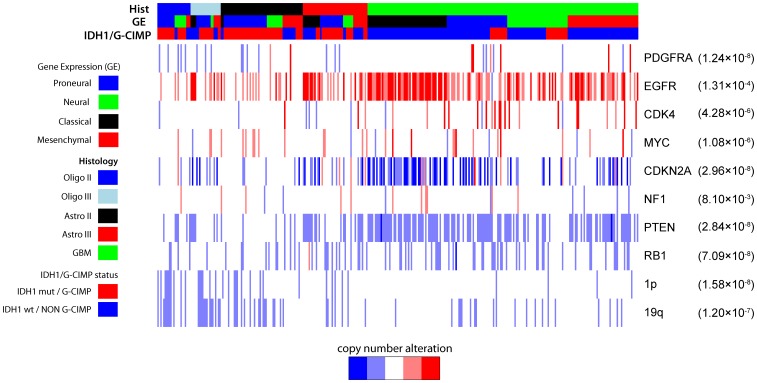
Somatic copy number analysis for Rembrandt dataset by histological group, gene expression subtype and IDH1/G-CIMP status (p values were accessed via fisher’s exact test).

To establish whether the reported associations between gene expression subtype and genomic characteristics could be similarly confirmed in GII/IIIs, we also analyzed DNA copy number profiles which were available for 334 samples in the Rembrandt data set. Codeletion of chromosomes 1p and 19qwas most frequent among *IDH1+/*G-CIMP Oligo II and Oligo III tumors compared with GBMs and *IDH1-/*non G-CIMP tumors (*P* = 1.58×10^−8^ for 1p and *P* = 1.20×10^−7^ for 19q, Figure 4). Within each histological group, the frequency of co-deletions was greater among *IDH1+/*G-CIMP tumors than *IDH1-/*non G-CIMP tumors, which is consistent with the Noushmehr et al findings. [30] *EGFR* amplification was observed at high frequency in classical tumors (Figure 4; *P* = 1.31×10^−4^), suggesting that *EGFR* amplification plays an important role in determining the classical gene expression signature. *EGFR* amplifciation, and *CDKN2A* deletions which frequently co-occur with gain of *EGFR*, consistently anti-correlated with *IDH1* wildtype status across both GII/III and GBMs, (*P* = 1.31×10^−4^ for *EGFR* and *P* = 2.96×10^−8^ for *CDKN2A*; Figure 4). Overall, the frequency of *CDK4/PDGFRA* amplification, markers for the proneural subtype, was found to be less than reported elsewhere in GBM [11] (Figure 4). *CDK4/PDGFRA* amplification was observed in proneural GBMs but not proneural GII/IIIs. *PTEN* deletions were more common in GBM than GII/III s, except classical grade II/III gliomas (*P* = 2.84×10^−8^ for *PTEN*). Genomic abnormalities of tumor suppressor *NF1*, which was reported as recurrently deleted and mutated in GBM, specifically the mesenchymal subtype, [11] were rare in the Rembrandt data set. This may be due to the limited coverage of *NF1* on the DNA copy number platform used for interrogation (Affymetrix 100k).

## Discussion

Recent advances in molecular classification of GBM raises the possibility of applying these novel classifications to grade II and III gliomas (GII/III). We have found that the Verhaak et al. [11] gene expression subtypes described for GBMs were applicable GII/IIIs of astrocytic and oligondendrocytic lineage using two published datasets (Rembrandt and JCO) and a newly derived dataset (DASL). In the Rembrandt dataset, we identified all four gene expression subtypes in GII/IIIs. Unsupervised cluster analysis identified four clusters that corresponded well with the original four gene expression based GBM subtypes, supporting generalizability of these gene expression subtype groups across all histological groups of gliomas. In the Rembrandt dataset the proportion of proneural, neural, classical and mesenchymal GBMs was similar to that reported for the TCGA GBM data in the Verhaak et al paper. [11] Grade II and III gliomas, however, displayed a distinct molecular subtype distribution, with a much larger proportion of proneural tumors. The proneural expression signature has been previously shown to best correspond to that of oligodendrocytes, [31] and the presence of the proneural signature may be a marker of preserved oligodendrocyte gene expression in GII/IIIs. In our analysis, proneural tumors had significantly longer survival than other groups of these tumors, which is consistent with prior reports for GBM [11], although not seen in a recent updated TCGA GBM analysis.^2^ Similar findings for the distribution of the gene expression based subtypes and survival for GBMs and GII/IIIs were validated in two additional datasets, a public dataset (JCO) and a newly generated dataset (DASL). We note that a relative small proportion (6.1%) of GBMs in the DASL data set was classified into the Neural subtype compared with our previous report (16%) [11]. But we have no reason to assume a technical bias introduced by the DASL platform rather than a natural fluctuation in tumor sampling, provided that 32 out of 35 cases with *IDH* R132 mutations were classified as Proneural in this data set.

The “classical” signature was rarely found in grade II tumors (astroctyomas or oligodendrogliomas), was slightly more common in grade III tumors, and was most frequent in GBMs. *EGFR* amplification was a key feature of the classical subtype in GBMs from TCGA, this finding is replicated in Rembrandt data, and importantly also found in classical GII/IIIs. This suggests that *EGFR* is a driver gene of the classical subtype regardless of histology. *CNKN2A* deletion frequently coexists with *EGFR* amplification in these classical tumors. Whether the classical subtype of GBMs are derived from this more uncommon subtype of GII/IIIs is unclear but it is possible that classical GBMs are primarily “de novo” GBMs/”pre GBMs” as has been previously proposed. [32] The identification of a classical signature in a GII/IIIs may be a sign of malignant potential.

The current study also replicated the original findings in GBM from Noushmehr et al, [13] using the epigenetically silenced gene expression signature of G-CIMP status as a surrogate for the DNA methylation signature. Moreover, recent findings in Turcan et al and Lu et al [15], [16] demonstrated that *IDH1* mutation is the molecular basis of G-CIMP in gliomas. An analysis by Lai et al. demonstrated that the most *IDH1* mutant tumors have a proneural subtype. [24] In this study, G-CIMP status was determined using gene expression array data for all 3 datasets, in order to utilize G-CIMP status as an informative surrogate for *IDH1* mutation status. *IDH1+/*G-CIMP status in GII/IIIs was significantly correlated with better prognosis among all subtypes across all 3 datasets, replicating the findings reported in Yan et al. [19] This finding also persisted when adjusting for age at diagnosis and gene expression subtype. Among GBMs, *IDH1+*/*G-CIMP* tumors also had a survival advantage, and survival was further improved among proneural GBMs with this status compared to *IDH1*-/non G-CIMP proneural tumors. Tumors with wild-type *IDH1* resemble GBM in outcome and gene expression profile. These findings further confirm that *IDH1* mutations are commonly reflective of favorable prognosis and are most commonly found in GII/IIIs.

A major challenge in investigating the gene expression patterns of GII/IIIs is these tumors are rare relative as compared to GBMs, and most publicly available datasets with high throughput data include few GII/III s. The JCO dataset had relatively few GII/IIIs. This may be due to differing inclusion/exclusion criteria between the datasets or differences in GBM prevalence among the different recruitment sites. The inclusion of two additional validation datasets, including one (DASL) with newly-collected samples and *IDH1* mutation status provides an effective and direct means to illustrate the relationships among *IDH1* mutation, proneural subtype and G-CIMP status. Although a strong and valid correlation between *IDH1* mutation, proneural subtype and G-CIMP positive status has been identified in several studies [15], [16], a few *IDH1* wildtype tumors in TCGA were classified as G-CIMP positive [14]. The Rembrandt database had less strict inclusion criteria than TCGA, and therefore the tumors were potentially more heterogeneous than TCGA. This might be a limitation for a discovery investigation, as the quality of the RNA may be questioned, but is considered a strength for a validation effort, such as this one, in which tumors more closely resemble those likely to be encountered in clinical practice. The ability to recognize gene expression and DNA methylation based subtypes in a less homogeneous set is encouraging for the future clinical applicability of these molecular subtype classifications.

*IDH1* also appears to be related to the copy number variation pattern of both GII/III s and GBMs. There appears to be three distinct types of GII/III s, those with and *IDH1* mutation and 1p and/or 19 q deletions (mostly oligodendrogliomas) [33], those with and *IDH1* mutation but no 1p/19q cytogenetic changes (further subdivided into whether they have *ATRX* or *CIC* and *FUBP1* mutations [34]) and *IDH1* wild-type GII/III s, which tend to have *EGFR* amplification and have been described as “pre-GBM” [34]. Our findings mirror this classification, with 1p/19q deletions mostly confined to oligodendrogliomas with and *IDH1* mutation or G-CIMP signature (Figure 4), and EGFR amplification observed mostly in *IDH1* wild type GII/III s and GBMs. Other copy number changes also seemed to be associated with *IDH1/*G-CIMP status. *CDK4* amplification was observed in proneural GBMs, but only if they also had an *IDH1* mutation or G-CIMP signature (Figure 4). *PTEN* deletions were fairly common across all gene expressions subtypes, but absent in *IDH1* mutant tumors. (Figure 4). *CDK4* and *PDGFRA* amplification were rare in GII/IIIs overall, suggesting that these events may play a role in the progression of a lower grade to a higher grade glioma. The frequency of *CDK4/PDGFRA* amplification was found to be less than reported elsewhere [11].

The results from the current study must be interpreted within the limitations of the study. Rembrandt and DASL lack DNA methylation data and therefore G-CIMP status was derived based on gene expression array data using five prediction models (probe sets of 10, 25, 50, 100 and 200) per Fine et al. [27] However survival and somatic copy number alteration patterns in this study are the same as those seen in the original G-CIMP study [13] suggesting that the G-CIMP classification can successfully be inferred from gene expression data. This is clinically advantageous, as it would obviate the need for DNA methylation specific studies in order to garner this important information which was confirmed in this study to be significantly associated with survival. In addition, the proportions for GBM and GII/IIIs with these data available were similar to that of the overall study sample. Similar patterns in survival were seen in this study as compared to the Verhaak et al [11] and Noushmehr et al papers. [13].

## Conclusion

Our findings suggest that gene-expression and DNA methylation based subtypes of GBMs are reproduced and applicable to grade II and III gliomas and have similar prognostic implications. In the progression from GII/III to GBM, the subtype spectrum changes from being dominated by proneural and neural tumors to increasingly more classical and mesenchymal tumors. Gaining an even more detailed understanding of the association between these GBM subtype classifiers, GII/III s and *IDH1* mutation/G-CIMP status could further our understanding of prognosis and disease progression and improve clinical management of this disease.

## Supporting Information

Figure S1 **Heatmap of 404 Rembrandt samples (N = 404) using Consensus clustering.**
(TIFF)

Table S1 **Cross tabulation for Consensus clustering results of gene expression subtype and histological group on 404 Rembrandt samples.**
(DOC)

Table S2 **Cross tabulation of histological groups and gene expression subtype on all non-TCGA samples (n = 690, which is 404 from Rembrandt+115 from DASL+171 from JCO; row percentages were shown).**
(DOC)

Table S3 **Distribution of gene expression subtypes and IDH1/G-CIMP status across Rembrandt, JCO and DASL datasets (p values were accessed via fisher’s exact test).**
(DOC)

Table S4 **Cross tab of gene expression subtypes, histological groups and IDH1/G-CIMP status on combination of Rembrandt, JCO and DASL (n = 690).**
(DOC)
