# Supplementary figures and images for: Molecular Subtypes of Glioblastoma Are Relevant to Lower Grade Glioma
Source: PLoS One. 2014 Mar 10;9(3):e91216. doi: 10.1371/journal.pone.0091216 (PMC3948818; doi:10.1371/journal.pone.0091216)

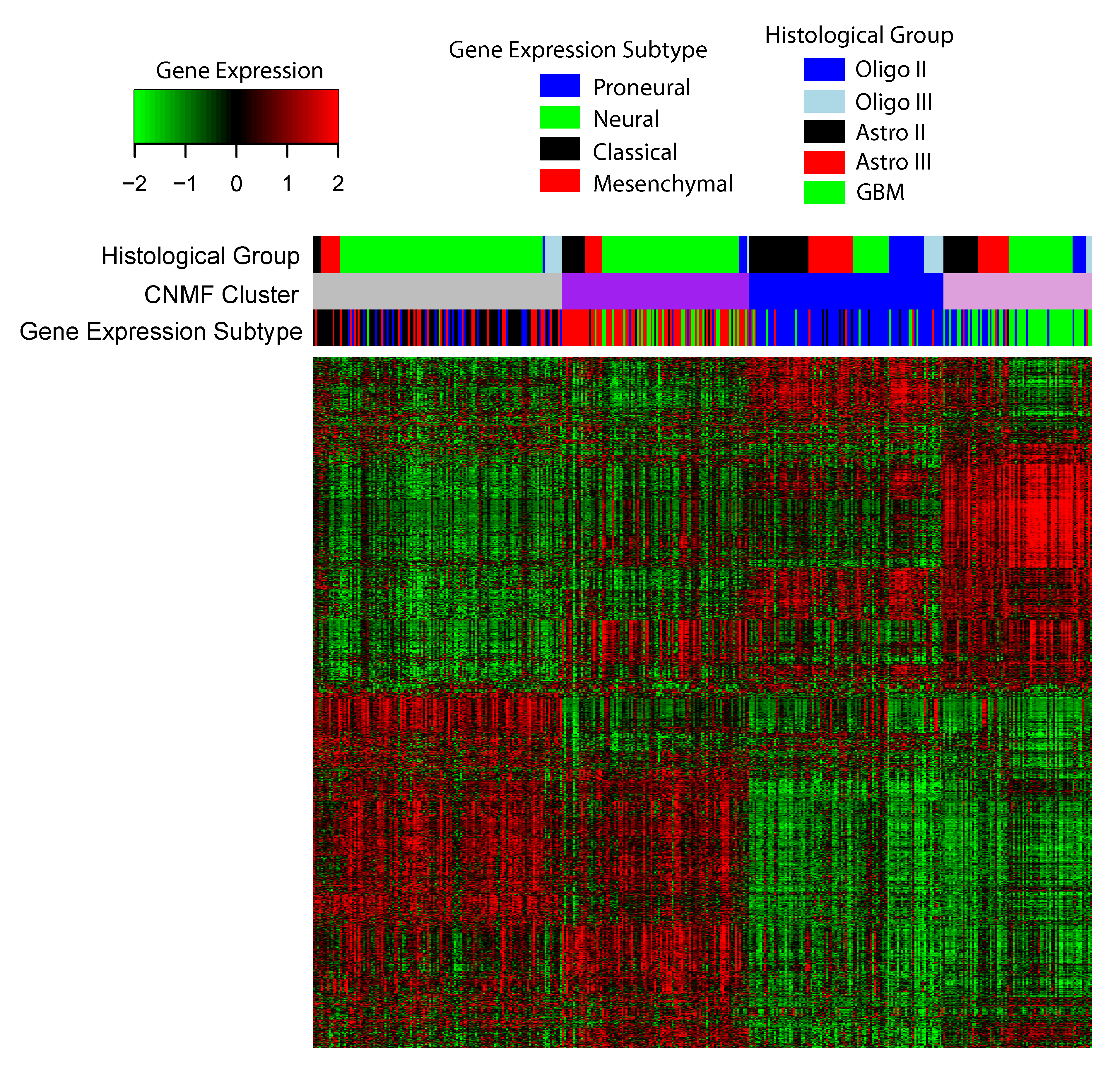

Supplement: Figure S1 — Heatmap of 404 Rembrandt samples (N = 404) using Consensus clustering. (TIFF) [file pone.0091216.s001.tiff]
