# Supplementary material for: Molecular Subtypes of Glioblastoma Are Relevant to Lower Grade Glioma
Source: PLoS One. 2014 Mar 10;9(3):e91216. doi: 10.1371/journal.pone.0091216 (PMC3948818; doi:10.1371/journal.pone.0091216)
Supplement: Table S1 — Cross tabulation for Consensus clustering results of gene expression subtype and histological group on 404 Rembrandt samples. (DOC) [file pone.0091216.s002.doc]

**Supplementary Table S1: Cross tabulation for Consensus clustering results of gene expression subtype and histological group** on 404 Rembrandt samples

|  | Gene expression subtype | | | | Histological Group | | | | |
| --- | --- | --- | --- | --- | --- | --- | --- | --- | --- |
|  | Proneural | Neural | Classical | Mesenchymal | Oligo II | Oligo III | Astro II | Astro III | GBM |
| **CNMF Cluster 1** | 26 | 2 | 73 | 28 | 1 | 9 | 4 | 10 | 105 |
| **CNMF Cluster 2** | 4 | 24 | 7 | 62 | 4 | 1 | 12 | 9 | 71 |
| **CNMF Cluster 3** | 79 | 8 | 7 | 7 | 18 | 10 | 31 | 23 | 19 |
| **CNMF Cluster 4** | 21 | 51 | 1 | 4 | 7 | 3 | 18 | 16 | 33 |
