# Supplementary material for: Molecular Subtypes of Glioblastoma Are Relevant to Lower Grade Glioma
Source: PLoS One. 2014 Mar 10;9(3):e91216. doi: 10.1371/journal.pone.0091216 (PMC3948818; doi:10.1371/journal.pone.0091216)
Supplement: Table S2 — Cross tabulation of histological groups and gene expression subtype on all non-TCGA samples (n = 690, which is 404 from Rembrandt+115 from DASL+171 from JCO; row percentages were shown). (DOC) [file pone.0091216.s003.doc]

**Supplementary Table S2: Cross tabulation of histological groups and gene expression subtype on all non-TCGA samples (n=690, which is 404 from Rembrandt+115 from DASL+171 from JCO; row percentages were shown).**

| **DASL (N=115)** | **Classical (N=26)** | **Mesenchymal (N=33)** | **Neural (N=9)** | **Proneural (IDH1+/G-CIMP)(N=31)** | **Proneural (IDH1-/NON G-CIMP)(N=16)** |
| --- | --- | --- | --- | --- | --- |
| GBM (N=79) | 25 (32 %) | 32 (41 %) | 4 (5 %) | 3 (4 %) | 15 (19 %) |
| ASTROII (N=5) | 0 (0 %) | 0 (0 %) | 1 (20 %) | 4 (80 %) | 0 (0 %) |
| ASTRO III (N=21) | 1 (5 %) | 1 (5 %) | 4 (19 %) | 15 (71 %) | 0 (0 %) |
| OLIGOII (N=4) | 0 (0 %) | 0 (0 %) | 0 (0 %) | 3 (75 %) | 1 (25 %) |
| OLIGOIII (N=6) | 0 (0 %) | 0 (0 %) | 0 (0 %) | 6 (100 %) | 0 (0 %) |
| ALL LGGs (N=36) | 1 (3%) | 1 (3%) | 5 (14%) | 28 (78%) | 1 (3%) |
| **JCO (N=171)** | **Classical (N=36)** | **Mesenchymal (N=52)** | **Neural (N=33)** | **Proneural (IDH1+/G-CIMP)(N=24)** | **Proneural (IDH1-/NON G-CIMP)(N=26)** |
| GBM (N=150) | 36 (24 %) | 49 (33 %) | 28 (19 %) | 14 (9 %) | 23 (15 %) |
| ASTRO III (N=21) | 0 (0 %) | 3 (14 %) | 5 (24 %) | 10 (48 %) | 3 (14 %) |
| **REMBRANDT (N=404)** | **Classical (N=88)** | **Mesenchymal (N=101)** | **Neural (N=85)** | **Proneural (IDH1+/G-CIMP)(N=86)** | **Proneural (IDH1-/NON G-CIMP)(N=44)** |
| GBM (N=228) | 64 (28 %) | 62 (27 %) | 55 (24 %) | 11 (5 %) | 36 (16 %) |
| ASTROII (N=65) | 3 (5 %) | 17 (26 %) | 11 (17 %) | 33 (51 %) | 1 (2 %) |
| ASTRO III (N=58) | 16 (28 %) | 12 (21 %) | 7 (12 %) | 20 (34 %) | 3 (5 %) |
| OLIGOII (N=30) | 1 (3 %) | 5 (17 %) | 10 (33 %) | 14 (47 %) | 0 (0 %) |
| OLIGOIII (N=23) | 4 (17 %) | 5 (22 %) | 2 (9 %) | 8 (35 %) | 4 (17 %) |
| ALL LGGs (N=176) | 24 (14%) | 39 (22%) | 30 (17%) | 75 (43%) | 8 (5%) |
| **TOTAL (N=690)** | **Classical**  **(N=150)** | **Mesenchymal**  **(N=186)** | **Neural**  **(N=127)** | **Proneural (IDH1+/G-CIMP)(N=141)** | **Proneural (IDH1-/NON G-CIMP)(N=86)** |
| GBM (N=457) | 125 (27%) | 143 (31%) | 87 (19%) | 28 (6%) | 74 (16%) |
| ASTRO II  (N=70) | 3 (4%) | 17 (24%) | 12 (17%) | 37 (53%) | 1 (1%) |
| ASTRO III  (N=100) | 17 (17%) | 16 (16%) | 16 (16%) | 45 (45%) | 6 (6%) |
| OLIGO II (N=34) | 1 (3%) | 5 (15%) | 10 (29%) | 17 (50%) | 1 (3%) |
| OLIGO III (N=29) | 4 (14%) | 5 (17%) | 2 (7%) | 14 (48%) | 4 (14%) |
| ALL LGGs (N=233) | 25 (11%) | 43 (18%) | 40 (17%) | 113 (48%) | 12 (5%) |
