# Supplementary material for: Molecular Subtypes of Glioblastoma Are Relevant to Lower Grade Glioma
Source: PLoS One. 2014 Mar 10;9(3):e91216. doi: 10.1371/journal.pone.0091216 (PMC3948818; doi:10.1371/journal.pone.0091216)
Supplement: Table S3 — Distribution of gene expression subtypes and IDH1/G-CIMP status across Rembrandt, JCO and DASL datasets (p values were accessed via fisher’s exact test). (DOC) [file pone.0091216.s004.doc]

**Supplementary Table S3: Distribution of gene expression subtypes and IDH1/G-CIMP status across** Rembrandt, JCO and DASL datasets (p values were accessed via fisher’s exact test).

|  | **Rembrandt** | **JCO** | **DASL** |  | **Fisher's exact P value** |
| --- | --- | --- | --- | --- | --- |
| **Classical** | 22% | 21% | 26% | Rembrandt vs JCO | 8.74 X 10 ^-1^ |
| **Mesenchymal** | 25% | 30% | 28% | Rembrandt vs DASL | 1.31X 10 ^-1^ |
| **Neural** | 21% | 19% | 9% | JCO vs DASL | 1.62X 10 ^-1^ |
| **Proneural** | 32% | 29% | 37% |  |  |
|  |  |  |  |  | **Fisher's exact P value** |
|  | **Rembrandt** | **JCO** | **DASL** | Rembrandt vs JCO | 6.50 X 10 ^-3^ |
| **IDH1-/Non G-CIMP** | 64% | 82% | 78% | Rembrandt vs DASL | 4.28X 10 ^-1^ |
| **IDH1+/G-CIMP** | 36% | 18% | 22% | JCO vs DASL | 7.75X 10 ^-2^ |
