# Supplementary material for: Molecular Subtypes of Glioblastoma Are Relevant to Lower Grade Glioma
Source: PLoS One. 2014 Mar 10;9(3):e91216. doi: 10.1371/journal.pone.0091216 (PMC3948818; doi:10.1371/journal.pone.0091216)
Supplement: Table S4 — Cross tab of gene expression subtypes, histological groups and IDH1/G-CIMP status on combination of Rembrandt, JCO and DASL (n = 690). (DOC) [file pone.0091216.s005.doc]

**Supplementary Table S4: Cross tab of gene expression subtypes, histological groups and IDH1/G-CIMP status on combination of** Rembrandt, JCO and DASL (n=690).

|  | **ASTRO II (N=70)** | | **ASTRO III (N=100)** | | **GBM (N=457)** | | **OLIGOII (N=34)** | | **OLIGOIII (N=29)** | |
| --- | --- | --- | --- | --- | --- | --- | --- | --- | --- | --- |
|  | **IDH1-/NON G-CIMP** | **IDH1+/G-GIMP** | **IDH1-/NON G-CIMP** | **IDH1+/G-GIMP** | **IDH1-/NON G-CIMP** | **IDH1+/G-GIMP** | **IDH1-/NON G-CIMP** | **IDH1+/G-GIMP** | **IDH1-/NON G-CIMP** | **IDH1+/G-GIMP** |
| **Classical (N=150)** | 3 | 0 | 14 | 3 | 123 | 2 | 0 | 1 | 4 | 0 |
| **Mesenchymal (N=186)** | 12 | 5 | 12 | 4 | 143 | 0 | 5 | 0 | 3 | 2 |
| **Neural (N=127)** | 3 | 9 | 8 | 8 | 63 | 24 | 2 | 8 | 0 | 2 |
| **Proneural (N=227)** | 1 | 37 | 6 | 45 | 74 | 28 | 1 | 17 | 4 | 14 |
